# The risk of early mortality in elderly patients with newly diagnosed acute myeloid leukemia

**DOI:** 10.1002/cam4.2740

**Published:** 2020-01-05

**Authors:** Chia‐Jen Liu, Ying‐Chung Hong, Ai Seon Kuan, Chiu‐Mei Yeh, Chun‐Kuang Tsai, Yao‐Chung Liu, Liang‐Tsai Hsiao, Hao‐Yuan Wang, Po‐Shen Ko, Po‐Min Chen, Jin‐Hwang Liu, Jyh‐Pyng Gau

**Affiliations:** ^1^ Division of Hematology and Oncology Department of Medicine Taipei Veterans General Hospital Taipei Taiwan; ^2^ Institute of Public Health National Yang‐Ming University Taipei Taiwan; ^3^ School of Medicine National Yang‐Ming University Taipei Taiwan; ^4^ Division of Hematology and Oncology Kaohsiung Veterans General Hospital Kaohsiung Taiwan; ^5^ Division of Neurosurgery Neurological Institute Taipei Veterans General Hospital Taipei Taiwan; ^6^ Institute of Biopharmaceutical Sciences National Yang‐Ming University Taipei Taiwan; ^7^ Chong Hin Loon Memorial Cancer and Biotherapy Research Center National Yang‐Ming University Taipei Taiwan

**Keywords:** acute myeloid leukemia, elderly, early mortality, epidemiology, prognostic models

## Abstract

**Background:**

Acute myeloid leukemia (AML) is a common hematologic neoplasm with high incidence and mortality in the elderly. Our aims were to explore risk factors for early mortality in elderly AML patients and develop a new prognostic score.

**Methods:**

We enrolled newly diagnosed AML patients age 60 and above at Taipei Veterans General Hospital between July 2008 and May 2017. The primary endpoint was early mortality, defined as death within two months after AML diagnosis. A multivariate Cox proportional hazards model was used to build a risk‐scoring system incorporating significant risk factors for AML.

**Results:**

The final cohort included 277 elderly AML patients. The median age was 74 (range 60‐96), and 61.7% were male. The two‐month mortality rate was 29.9%. Age ≥ 80 (adjusted HR 1.88), myocardial infarction (adjusted HR 1.87), ECOG ≥ 2 (adjusted HR 2.10), complex karyotype (adjusted HR 3.21), bone marrow blasts ≥ 70% (adjusted HR 1.88), WBC ≥ 100 × 10^9^/L (adjusted HR 3.31), and estimated glomerular filtration rate (eGFR) < 45 (adjusted HR 2.60) were identified as independent predictors for early mortality in the multivariate analysis. A simplified score incorporating the seven factors was developed with good predictive ability measured by Harrell's C statistic [0.72 (95% CI 0.66‐0.78)].

**Conclusions:**

We identified seven potential risk factors for early mortality and built up a new prognostic score for elderly AML patients. The new score may help clinicians stratify patients and initiate appropriate management. Further validation of our findings on other cohorts is warranted.

## Graphical abstract

Age ≥ 80, myocardial infarction, ECOG ≥ 2, complex karyotype, BM blasts ≥ 70%, WBC ≥ 100 × 10^9^/L, and eGFR < 45 were seven risk factors for early mortality in elderly AML. A new prognostic score for elderly AML patients may help clinicians stratify patients and initiate appropriate management.

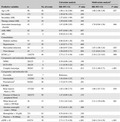

## 1 INTRODUCTION

Acute myeloid leukemia (AML) is a hematological neoplasm commonly seen in Taiwan and many other countries.^1^, ^2^ The estimated number of new cases was 21450 in the US in 2019.^2^ The median age of AML patients at diagnosis is about 70 years old.^3^ The median progression‐free survival (PFS) and overall survival (OS) of elderly AML patients (age ≥ 60) receiving standard induction treatment (7 + 3, cytarabine with an anthracycline) were 6.7 and 14.7 months, respectively.^4^ In elderly patients receiving hypomethylating agents (HMAs), the median PFS and OS were only 4.1 and 4.3 months, respectively. The high mortality in elderly AML patients was reported to be associated with old age, poor performance status, and disease characteristics such as high‐risk cytogenetics and complex karyotypes.^5^, ^6^, ^7^, ^8^

Because the characteristics and outcomes of AML are very heterogeneous, several risk stratification systems have been developed. Malfuson et al reviewed 416 elderly patients treated in the ALFA‐9803 trial in France and identified high‐risk cytogenetics and the presence of at least two of the three factors (age ≥ 75, performance status ≥ 2, white blood cell (WBC) ≥50 × 10^9^/L) attributed to OS.^5^ Wheatley et al analyzed 2483 AML patients age ≥ 60 enrolled in two UK trials—the Medical Research Council AML11 Trial and the Leukaemia Research Fund AML14 Trial.^6^ They built up a risk score using cytogenetics, WBC, performance status, age, and AML type. Kantarjian et al found that intensive chemotherapy did not benefit most elderly AML patients (age ≥ 70). They identified age ≥ 80, complex karyotypes, poor performance and elevated creatinine > 1.3 mg/dL as independent predictors for eight‐week mortality.^7^ Walter et al reviewed 2238 adults treated at MD Anderson Cancer Center and 1127 patients from 10 SWOG trials. They found that old age, poor performance status, and low platelet count were risk factors of early death after induction therapy for newly diagnosed AML.^8^ Ramos et al conducted a retrospective study (ALMA Registry) in Spain and developed the European ALMA score (using ECOG, WBC, and cytogenetics) to predict OS of unfit AML patients treated with an HMA.

The scoring systems mentioned above were developed in the US and European countries. They might not be suitable for predicting outcomes of our population due to differences in ethnicity and healthcare systems. We hence investigated risk factors for early mortality in elderly AML patients and aimed to develop a new scoring system, combining clinical data and genetic abnormalities, to predict outcomes of elderly AML patients in Asian populations.

## 2 MATERIALS AND METHODS

### 2.1 Study population

This study included consecutive elderly patients with newly diagnosed AML between 1 July 2008 and 31 May 2017 at Taipei Veterans General Hospital. Follow‐up was continued to 31 July 2017. AML was diagnosed based on WHO criteria,^9^ and bone marrow (BM) examinations were performed at diagnosis. Patients younger than 60 and those without a pathologic diagnosis were excluded.

### 2.2 Data collection and study endpoint

Data collection was performed by reviewing medical records. The following clinical characteristics were obtained: age, sex, height, weight, and smoking status; comorbidities, including diabetes mellitus, hypertension, myocardial infarction, and ulcer diseases; laboratory parameters, including white blood cells with differential counts, hemoglobin, platelets, and blasts in peripheral blood and BM; performance status according to the Eastern Cooperative Oncology Group performance score (ECOG)^10^, ^11^; genetic risk status, including cytogenetics and molecular abnormalities^12^; which was recorded at diagnosis. Having an antecedent hematologic disorder was defined as having a history of specifically myelodysplastic syndromes, myeloproliferative neoplasms, or aplastic anemia.^13^ Cutoff values for age, performance status, WBC, platelet, cytogenetics, creatinine, and AML type were chosen according to those of the previous studies.^5^, ^6^, ^7^, ^8^, ^14^, ^15^, ^16^ The estimated glomerular filtration rate (eGFR) was calculated based on Chronic Kidney Disease Epidemiology Collaboration Equations^17^, ^18^

Information on treatment regimens composed of induction and consolidation treatment was collected. Intensive treatment was defined as cytarabine with an anthracycline treatment or high‐dose cytarabine. Supportive care was defined as low‐dose cytarabine, hydroxyurea, or no AML‐specific treatment with blood transfusion.

Our primary endpoint was early mortality, defined as death within 60 days after AML initial diagnosis.^19^ Our retrospective review of medical records was conducted in accordance with the institutional ethics committee and in agreement with the Helsinki Declaration of 1975, revised in 2008. This study was approved by the Institutional Review Board at Taipei Veterans General Hospital (No. 2019‐05‐009BC).

### 2.3 Statistical analysis

Patients’ demographic and clinical characteristics were presented as the total number (*n*) and proportion (%) for categorical data, and medians and interquartile ranges (IQR) for continuous data (skewed).

Data for patients who did not have early mortality or who were lost to follow‐up were censored. In the survival analysis, the Kaplan‐Meier method was used for estimation of cumulative incidence of early mortality, and differences between groups were tested by log‐rank test. Hazard ratios (HRs) and 95% confidence intervals (CIs) were calculated using Cox proportional hazards models, controlling for potential confounding factors in the multivariate model. We first used a univariate model to identify potential risk factors for AML early mortality. All risk factors with *P* < .1 in the univariate model were further entered into the multivariate analysis. All independent risk factors identified in the multivariate analysis were then used to build a predictive model of early mortality.

The *β*‐coefficients of all significant risk factors in the multivariate Cox proportional hazards model were used to build a new risk‐scoring system. We also built a simplified score by assigning one point to each significant variable. Model discrimination was estimated by Harrell's C statistics. The discriminatory ability of our score and existing prognostic scores identified by a systematic review were compared using the Akaike information criterion (AIC) and Bayesian information criterion (BIC) calculations.

Data management and all statistical analysis were performed using SAS 9.4 software (SAS Institute Inc) and STATA statistical software, version 15.1 (StataCorp). All statistically significant levels were set at *P* < .05.

## 3 RESULTS

### 3.1 Clinical characteristics of the study population

A total of 478 patients with newly diagnosed AML between 1 July 2008 and 31 May 2017 at Taipei Veterans General Hospital were identified. Patients who had no pathological confirmation (n = 5) and those diagnosed at age < 60 (n = 196) were excluded. Finally, 277 elderly AML patients were enrolled in the study. The median age was 74 (range 60‐96), and 61.7% were men. Ninety‐four patients (33.9%) had secondary AML. Hypertension (45.9%) and diabetes mellitus (33.6%) were the most common comorbidities. In regards to cytogenetics and molecular abnormalities, 13.0%, 48.0%, and 33.9% had favorable, intermediate, and poor/adverse risk, respectively. The median of BM blasts was 80%, and 66.2% of the patients also had the presence of blasts in their peripheral blood. The initial treatment was categorized into cytarabine‐based intensive treatment, azacitidine or decitabine, all‐trans retinoic acid, and supportive care, which were 32.1%, 18.8%, 2.9%, and 46.2% of 277 patients, respectively (Table 1).

**Table 1:** Baseline characteristics of elderly acute myeloid leukemia patients

Characteristics	Total n = 277
Median age, years (range)	74 (60‐96)
≥80	96 (34.7)
<80	181 (65.3)
Secondary AML	94 (33.9)
Therapy‐related AML	25 (9.0)
Antecedent hematologic disorder	42 (15.2)
AML‐MRC	82 (29.6)
APL	11 (4.0)
Comorbidities	
Diabetes mellitus	93 (33.6)
Hypertension	127 (45.9)
Myocardial infarction	54 (19.5)
Ulcer disease	45 (16.3)
ECOG	
0‐1	154 (55.6)
≥2	116 (41.9)
Unknown	7 (2.5)
Cytogenetics and molecular abnormalities	
NPM1	20/125 (16.0)
FLT3‐ITD	18/125 (14.4)
Complex karyotype	58/263 (22.1)
Cytogenetics and molecular risk status	
Favorable	36 (13.0)
Intermediate	133 (48.0)
Poor/adverse	94 (33.9)
Unknown	14 (5.1)
Lab data, median (IQR)	
Bone marrow blast, %	80 (40‐90)
Presence of blasts in peripheral blood	182/275 (66.2)
White blood cell count,/uL	5830 (1890‐36 600)
Absolute neutrophil count,/uL	1254.4 (325.0‐5072.0)
Hemoglobin, g/dL	8.4 (7.4‐9.5)
Platelets,/uL	50 000 (26 000‐86 000)
Creatinine, mg/dL	1.0 (0.8‐1.4)
eGFR	65.2 (45.9‐82.8)
Initial treatment	
Cytarabine‐based intensive treatment	89 (32.1)
Azacitidine or decitabine	52 (18.8)
All‐trans retinoic acid	8 (2.9)
Supportive care	128 (46.2)

Abbreviations: AML, acute myeloid leukemia; AML‐MRC, acute myeloid leukemia with myelodysplasia‐related changes; APL, acute promyelocytic leukemia; ECOG, Eastern Cooperative Oncology Group performance; eGFR, estimated Glomerular filtration rate; IQR, interquartile range.

### 3.2 Risk factors of early mortality

**Figure 1:**
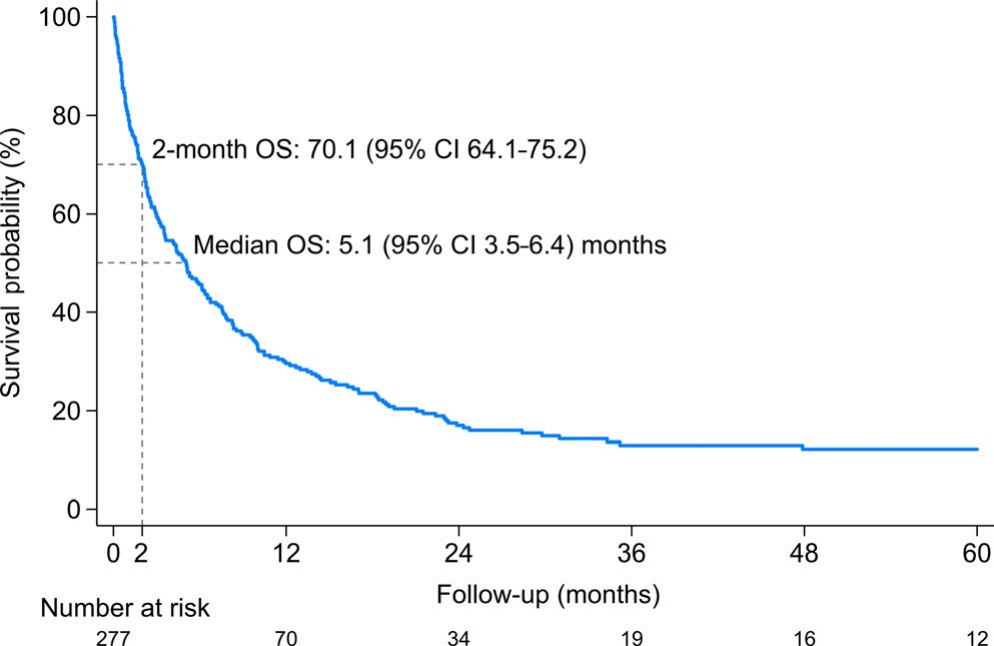
Overall survival of elderly patients with newly diagnosed acute myeloid leukemia

The median OS was 5.1 (95% CI 3.5‐6.4) months from diagnosis of AML. The probability of survival within 2 months was 70.1% (95% CI 64.1‐75.2%). The cumulative probability of the death curve is shown in Figure 1. In the univariate analysis, we found that age ≥ 80, having an antecedent hematologic disorder, myocardial infarction, ulcer diseases, ECOG performance status ≥ 2, complex karyotype, BM blasts ≥ 70%, WBC count ≥ 100 × 10^9^/L, and eGFR < 45 were associated with early mortality in patients with AML (Table 2). In the multivariate analysis, age ≥ 80 (adjusted HR 1.88, 95% CI 1.08‐3.30), myocardial infarction (adjusted HR 1.87, 95% CI 1.08‐3.24), ECOG ≥ 2 (adjusted HR 2.10, 951.22‐3.63), complex karyotype (adjusted HR 3.21, 95% CI 1.80‐5.71), BM blasts ≥ 70% (adjusted HR 1.88, 95% CI 1.07‐3.32), WBC ≥ 100 × 10^9^/L (adjusted HR 3.31, 95% CI 1.59‐6.90), and eGFR < 45 (adjusted HR 2.60, 95% CI 1.54‐4.39) remained statistically significant (Table 2).

**Table 2:** Risk factors for early mortality in elderly patients with newly diagnosed acute myeloid leukemia

Predictive variables	n	No. of events	Univariate analysis		Multivariate analysis^a^	
			HR (95% CI)	*P* value	HR (95% CI)	*P* value
Age ≥ 80	96	36	1.80 (1.16‐2.80)	.009	1.88 (1.08‐3.30)	.027
Sex (male)	171	52	1.20 (0.76‐1.91)	.436		
Secondary AML	94	31	1.27 (0.81‐1.99)	.307		
Therapy‐related AML	25	10	1.59 (0.82‐3.09)	.169		
Antecedent hematologic disorder	42	17	1.67 (0.98‐2.85)	.062	1.78 (0.96‐3.30)	.066
AML‐MRC	82	24	0.97 (0.60‐1.56)	.897		
APL	11	2	0.58 (0.14‐2.36)	.446		
Comorbidities						
Diabetes mellitus	93	23	0.80 (0.49‐1.30)	.370		
Hypertension	127	38	1.10 (0.71‐1.72)	.660		
Myocardial infarction	54	21	1.60 (0.97‐2.64)	.065	1.87 (1.08‐3.24)	.025
Ulcer disease	45	17	1.58 (0.92‐2.71)	.094	1.21 (0.65‐2.24)	.553
ECOG ≥ 2	116/270	46	2.73 (1.71‐4.38)	<.001	2.10 (1.22‐3.63)	.008
Cytogenetics and molecular abnormalities						
NPM1	20/125	2	0.39 (0.09‐1.64)	.199		
FLT3‐ITD	18/125	4	1.11 (0.39‐3.22)	.841		
Complex karyotype	58/263	22	1.90 (1.15‐3.14)	.013	3.21 (1.80‐5.71)	<.001
Cytogenetics and molecular risk						
Favorable	36/263	7	Reference			
Intermediate	133/263	36	1.44 (0.64‐3.25)	.374		
Poor/adverse^b^	94/263	28	1.72 (0.75‐3.93)	.201		
Lab data						
Bone marrow blasts ≥ 70%	157/263	50	1.65 (1.00‐2.73)	.049	1.88 (1.07‐3.32)	.029
Presence of blasts in peripheral blood	182/275	59	1.47 (0.89‐2.44)	.135		
White blood cell count ≥ 100 K/µL	26	15	3.19 (1.82‐5.62)	<.001	3.31 (1.59‐6.90)	.001
Absolute neutrophil count < 500/µL	82	19	0.72 (0.43‐1.20)	.209		
Hemoglobin < 10 g/dL	231	63	0.76 (0.44‐1.31)	.320		
Platelets < 20 000/µL	45	16	1.45 (0.84‐2.51)	.184		
eGFR < 45	63/274	30	2.71 (1.72‐4.28)	<.001	2.60 (1.54‐4.39)	<.001

Abbreviations: AML, acute myeloid leukemia; AML‐MRC, acute myeloid leukemia with myelodysplasia‐related changes; APL, acute promyelocytic leukemia; CI, confidence interval; ECOG, Eastern Cooperative Oncology Group performance; eGFR, estimated Glomerular filtration rate; HR, hazard ratio.

a All factors with *P* < .1 in the univariate analysis were included in the Cox multivariate analysis.

b Poor/adverse risk was defined as complex (≥3 clonal chromosomal abnormalities), monosomal karyotype, −5, 5q‐, −7, 7q‐, 11q23 ‐ non t(9;11), inv(3), t(3;3), t(6;9), t(9;22), or FLT3‐ITD mutation with wild‐type NPM1.

### 3.3 Risk stratification for elderly AML patients

We built a prognostic model incorporating all independent risk factors. The *β*‐coefficients of all significant variables in the multivariate analysis were used to create the prognostic index for early mortality. The resulting equation is as follows:

$$\begin{array}{cc} \text{Index}\; & =\;0.63\times \left[\text{age}\geq 80\right]+0.63\times \left[\text{myocardial infarction}\right]\\ & \;+0.74\times \left[\text{ECOG}\geq 2\right]+1.16\times \left[\text{complex karyotype}\right]\\ & \;+0.63\times \left[\text{bone marrow blasts}\geq 70\%\right]+1.20\\ & \;\times \left[\text{WBC count}\geq 100\times 10^{9}/L\right]+0.96\times \left[\text{eGFR}<45\right] \end{array}$$

The median index in all participants was 1.4 (IQR 0.6‐2.3). Each increment in the index was associated with a nearly three‐times increase in hazard for early mortality (HR 2.70, 95% CI 2.06‐3.53). The prognostic index discriminated the risk of early mortality in elderly AML patients with an estimated Harrell's C statistic of 0.74 (95% CI 0.68‐0.81). A simpler risk model may be easier to use in clinical practice. Thus, we defined a simplified prognostic model by assigning one point for each of the seven independent predictors (age ≥ 80, myocardial infarction, ECOG ≥ 2, complex karyotype, BM blasts ≥ 70%, WBC ≥ 100 × 10^9^/L, and eGFR < 45). Then we divided the patients into low‐risk (score 0‐1), intermediate‐risk (score 2‐3), and high‐risk (score 4‐5) groups, based on the scoring of the simplified model. The numbers of patients belonging to low‐, intermediate‐, and high‐risk groups were 91 (37.0%), 118 (48.0%), and 37 (15.0%), respectively (Table 3).

**Table 3:** Incidence of early mortality in acute myeloid leukemia patients with risk scoring

Risk score	Level	n	No. of events	2‐mo mortality rate	HR (95% CI)	*P*‐value	AIC	BIC	Harrell C statistics
Prognostic index^a^		246	64	27.1 (21.8‐33.3)	2.70 (2.06‐3.53)	<.001	624.82	626.97	0.74 (0.68‐0.81)
Simplified risk score^b^	0‐1	91	9	10.3 (5.5‐18.8)	Reference		635.66	639.97	0.72 (0.66‐0.78)
	2‐3	118	30	26.8 (19.4‐36.2)	3.01 (1.43‐6.33)	.004			
	4‐6	37	25	70.7 (55.1‐84.8)	12.04 (5.60‐25.92)	<.001			

Abbreviations: AIC, Akaike information criterion; BIC, Bayesian information criterion; CI, confidence interval; HR, hazard ratio.

a Index = 0.63 × [age ≥ 80] + 0.63 × [myocardial infarction] + 0.74 × [ECOG ≥ 2] + 1.16 × [complex karyotype] + 0.63 × [bone marrow blasts ≥ 70%] + 1.20 × [WBC count ≥ 100 × 10^9^/L] + 0.96 × [eGFR < 45].

b Simplified risk score = [age ≥ 80] + [myocardial infarction] + [ECOG ≥ 2] + [complex karyotype] + [bone marrow blasts ≥ 70%] + [WBC count ≥ 100 × 10^9^/L] + [eGFR < 45].

**Figure 2:**
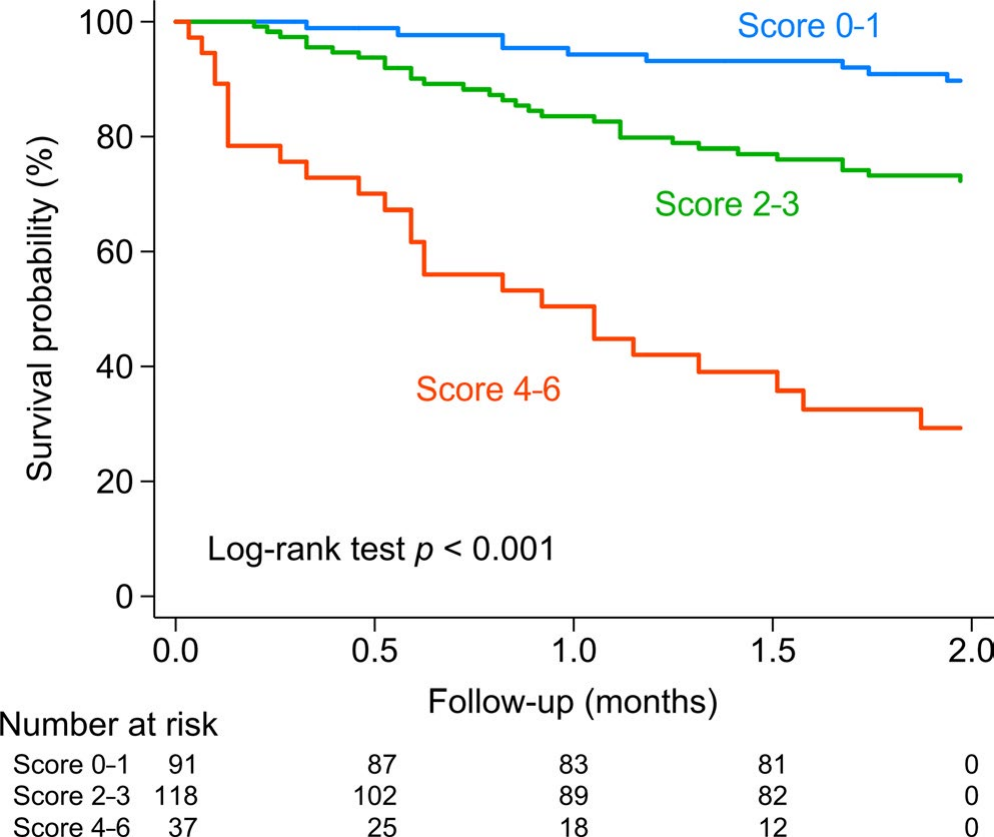
Kaplan‐Meier estimates in acute myeloid leukemia patients stratified by risk scoring. We specified the risk strata by assigning one point for each of the seven factors (age ≥ 80, myocardial infarction, ECOG ≥ 2, complex karyotype, bone marrow blasts ≥ 70%, WBC count ≥ 100 K/µL, and eGFR < 45) in a scoring system

The Kaplan‐Meier curves demonstrate that patients with higher scores had significantly shorter survival (log‐rank test *P* < .001) (Figure 2). The HR for the intermediate‐ and high‐risk groups were 3.01 (95% CI 1.43‐6.33) and 12.04 (95% CI 5.60‐25.92), respectively, when compared with the low‐risk group. The predictive ability of the simplified model measured by Harrell's C statistic was 0.72 (95% CI 0.66‐0.78).

### 3.4 Comparisons with different scoring systems

We systematically reviewed existing prognostic models for elderly AML. We found five scoring models that do not require additional specific examinations and later applied them to our elderly AML cohort. Supplemental Table 2 lists the performance of our model and the other five models. The median Malfuson index was 9.5 (IQR 5.8‐39.7). The index could not predict early mortality in our cohort.^5^ However, the simple decisional index had better performance (HR 3.03, 95% CI 1.72‐5.32 for score 1 and 3.19, 95% CI 1.49‐6.81 for score 2, respectively). The Wheatley index and its simplified risk score identified the poor‐risk group with a significant higher risk of early mortality (HR 3.43, 95% CI 1.47‐7.96 for index and HR 3.49, 95% CI 1.40‐8.71 for simplified risk score, respectively) although the probability of early mortality was similar between good and standard risk groups.^6^ Kantarjian's prognostic model divided the patients into four groups.^7^ The patients with two or ≥ three adverse factors had significantly high risk of early mortality (HR 3.35, 95% CI 1.53‐7.31 for score 2 and 7.08, 95% CI 3.22‐15.58 for score ≥ 3 adverse factors, respectively). Another two scoring models purposed by Ramos, Walter, et al predicted early mortality in the high‐risk group but had no significant difference between low‐ and intermediate‐risk groups.^8^, ^14^ Our prognostic model had the highest Harrell's C statistic and the lowest AIC and BIC compared with the other five prognostic models.

### 3.5 Causes of early mortality

**Figure 3:**
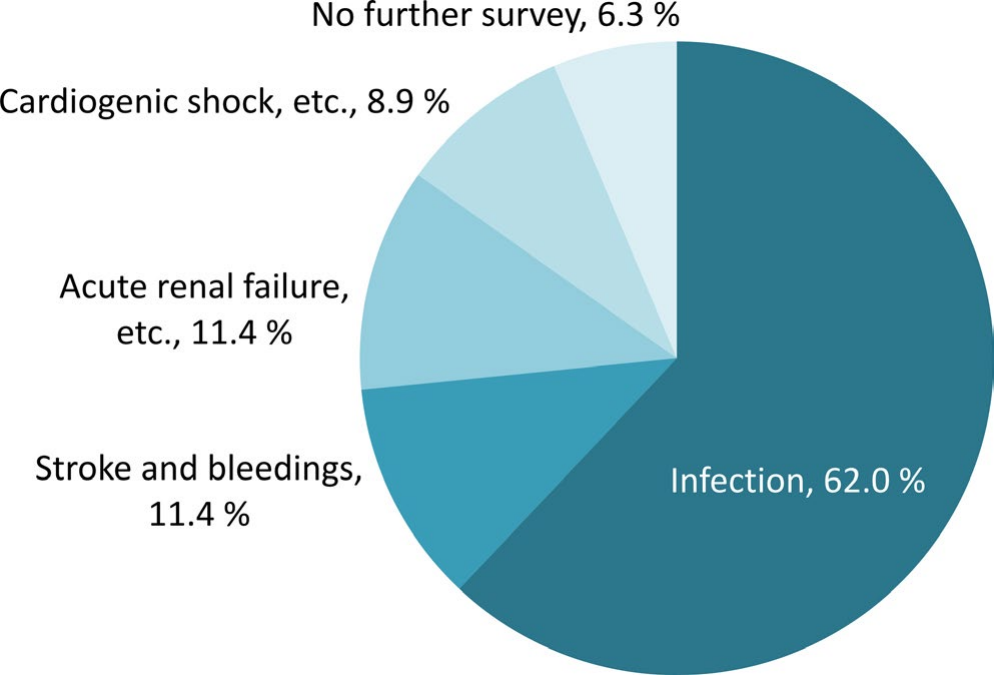
Direct causes of early mortality. Infection, including pneumonia, bacteremia, and others; acute renal failure, etc, including acute renal failure, tumor lysis, or electrolyte imbalance; cardiogenic shock, etc, including cardiogenic shock or pulmonary edema

The direct causes of early death that occurred during this study are summarized in Figure 3. The most common cause was infection (49 patients, 62.0%). Prolonged neutropenia contributed to pneumonia, septic shock, and acute respiratory failure in patients who died early of infection. Five patients had ischemic strokes or intracranial hemorrhage; other bleeding causes included two patients with pulmonary hemorrhage and two patients with massive gastrointestinal bleeding. Acute renal failure occurred in nine patients, in which eight cases had acute kidney injuries and/or tumor lysis syndrome, and one case had central diabetes insipidus due to brain infiltration associated with acute leukemia cells. Cardiogenic shock occurred in four patients; three patients died of acute pulmonary edema without heart evaluation. Sudden death occurred in one patient at AML diagnosis. Four patients died at home or at other institutions and were without detailed information.

## 4 DISCUSSION

To the best of our knowledge, this is the first study that predicts early mortality of elderly AML patients in Asia and validates other prognostic models developed in Western countries. Based on the prognostic factors, we classified patients into three risk groups. The early mortality rate of low‐, intermediate‐, and high‐risk groups were 10.3%, 26.8%, and 70.7%, respectively. Our results may help physicians stratify patients and initiate proper treatment.

We have systematically reviewed existing AML prognostic scoring systems and evaluated the five prognostic models in our elderly AML cohort. Those AML risk‐stratified models used common risk factors, including age,^5^, ^6^, ^7^, ^8^ performance status,^6^, ^7^, ^8^, ^14^ WBC,^5^, ^6^, ^14^, ^15^ platelet,^8^ creatinine,^7^ BM blasts,^14^ cytogenetics,^5^, ^6^, ^7^ and types of AML.^6^ Our model consists of age, myocardial infarction, ECOG, complex karyotype, BM blasts, WBC, and eGFR, which are a part of routine workup for AML at diagnosis. Our model can easily be applied to AML patients in daily practice. In addition, our model had the highest C statistic and the lowest AIC and BIC, in comparison with other prognostic models.

We found that age is a key element of our model and some other models.^5^, ^6^, ^7^, ^8^ Elderly patients frequently have multiple comorbidities and have a lower probability of responding to induction treatment.^5^ Although our model has adjusted for some age‐associated adverse characteristics such as secondary AML and complex karyotypes, old age still negatively impacts patient outcomes.^6^ Kantarjian et al compared the outcomes of elderly AML patients stratified by age. They found age ≥80 years was an independent adverse prognostic factor for eight‐week mortality.^7^, ^20^ Farag et al reported that being age 80 or older doubled the risk of death for AML patients.^21^ Performance status is also an important element of disease prognosis, and it was included in all five existing prognostic models and our model. Poor performance status reflects organ dysfunctions.^16^, ^22^ Several studies show that poor performance status is the strongest predictor for OS and treatment‐related mortality.^5^, ^8^

High WBC counts were associated with coagulopathy, pulmonary and CNS leukostasis, and renal failure.^20^, ^23^, ^24^, ^25^ Valcarcel et al found that leukocytosis (>100 × 10^9^/L) doubled the risk of death during standard induction chemotherapy in newly diagnosed AML patients.^15^ A systemic review and meta‐analysis shows that early mortality related to hyperleukocytosis in AML is not reversed by leukapheresis or pharmacologic cytoreduction.^26^ Acute renal failure was a common cause of death in previous studies.^15^, ^26^, ^27^ Low creatinine clearance may increase the toxicity of chemotherapy and other medication.^28^ It also contributes to tumor lysis syndrome.^29^, ^30^, ^31^ Therefore, drug dose adjustment is needed in patients with renal impairment.^32^ In the current study, we use eGFR because creatinine differs by sex.^17^, ^18^ Myeloblasts in BM reflect disease burden. Farag et al found a 6% increase of mortality risk with every 1% increase of BM blasts. Complex karyotype is an unfavorable prognostic factor in AML patients. Many studies have shown that complex karyotype can predict a lower chance of achieving CR or post‐induction mortality in elderly AML patients.^7^, ^16^, ^21^ Therefore, elderly AML patients with unfavorable cytogenetics might not benefit from standard therapies.^33^ Myocardial infarction is a key prognostic factor for cancer patients.^34^ Anthracycline treatment might cause cardiomyopathy and heart failure.^35^ The patients with myocardial infarction received less intensive treatment in the current study.

A remarkable study from MD Anderson Cancer Center in 2010 revealed that intensive chemotherapy did not benefit most elderly AML patients.^7^ In the study, four of the variables (age, performance status, karyotype, and creatinine) in our model were used to predict the probability of early mortality after receiving intensive chemotherapy. The median survival of elderly AML patients with one, two, and ≥ three risk factors were only 5.3, 1.5, and 0.5 months, respectively. Therefore, they recommend elderly patients with any risk factor not receive intensive chemotherapy.

Our study has some limitations. Our patients did not receive new tests for molecular abnormalities, such as *ASXL1*, *TP53*, and *RUNX1*, which were recommended by the European LeukemiaNet guidelines in 2017.^12^ The choice of AML treatment was decided based on patient characteristics and was highly associated with all prognostic factors. Receiving supportive care might be a potential confounding factor in this study. Due to the retrospective nature of this study, our findings may be subject to selection bias, so further validations are warranted.

## 5 CONCLUSION

Early mortality in elderly AML patients is still common despite the development of novel therapies. It's crucial to find out the prognostic factors and plan management strategies according to disease risk stratification. Of great importance, we systemically reviewed all current prognostic models and include risk factors suitable for routine practice. We identified seven risk factors of early mortality, including age ≥ 80, myocardial infarction, ECOG ≥ 2, complex karyotype, BM blasts ≥ 70%, WBC ≥ 100 × 10^9^/L, and eGFR < 45. Our findings may help clinicians stratify elderly AML patients and initiate appropriate treatment.

## COMPETING INTERESTS

The authors declare no conflict of interest.

## AUTHORS’ CONTRIBUTIONS

C‐JL had full access to all of the data in the study and takes responsibility for the integrity of the data and the accuracy of the data analysis. C‐JL, Y‐CH, and C‐KT designed the study. C‐MY and C‐JL acquired the data and performed statistical analysis. C‐JL, C‐MY, and C‐KT provided the final interpretation of the results. C‐JL and C‐MY drafted the manuscript. Y‐CH, A‐SK, Y‐CL, L‐TH, H‐YW, and P‐SK made critical revisions to the manuscript for important intellectual content. C‐JL, C‐KT, and C‐MY provided administrative, technical, and material support. P‐MC, J‐HL, and J‐PG were the study supervisors. C‐JL and J‐PG act as guarantors and accept responsibility for the integrity of the work as a whole. All authors have read and approved the final manuscript.

## Data Availability

Supplementary information and data are available at Cancer Medicine’s website.
