# Supplementary material for: The risk of early mortality in elderly patients with newly diagnosed acute myeloid leukemia
Source: Cancer Med. 2020 Jan 5;9(4):1572–80. doi: 10.1002/cam4.2740 (PMC7013048; doi:10.1002/cam4.2740)
Supplement: Supplementary file 1 [file CAM4-9-1572-s001.docx]

**Supplemental Material**

**Supplemental Table 1 Baseline patient characteristics of acute myeloid leukemia with and without early mortality**

| **Characteristics** | **Total**  ***n* = 277** | **Early mortality**  ***n* = 79** | **Non-early mortality**  ***n* = 198** | ***P* value** |
| --- | --- | --- | --- | --- |
| Median age, years (range) | 74 (60–96) | 78 (60–93) | 72 (65–81) | 0.002 |
| ≥ 80 | 96 (34.7) | 36 (45.6) | 60 (30.3) | 0.016 |
| < 80 | 181 (65.3) | 43 (54.4) | 138 (69.7) |  |
| Sex (male) | 171 (61.7) | 52 (65.8) | 119 (60.1) | 0.376 |
| Secondary AML | 98 (35.4) | 34 (43.0) | 64 (32.3) | 0.239 |
| Therapy-related AML | 25 (9.0) | 10 (12.7) | 15 (7.6) | 0.183 |
| Antecedent hematologic disorder | 42 (15.2) | 17 (21.5) | 25 (12.6) | 0.062 |
| AML-MRC | 82 (29.6) | 24 (30.4) | 58 (29.3) | 0.858 |
| APL | 11 (4.0) | 2 (2.5) | 9 (4.6) | 0.734 |
| Comorbidities |  |  |  |  |
| Diabetes mellitus | 93 (33.6) | 23 (29.1) | 70 (35.4) | 0.321 |
| Hypertension | 127 (45.9) | 38 (48.1) | 89 (45.0) | 0.635 |
| Myocardial infarction | 54 (19.5) | 21 (26.6) | 33 (16.7) | 0.060 |
| Ulcer disease | 45 (16.3) | 17 (21.5) | 28 (14.1) | 0.133 |
| ECOG |  |  |  |  |
| 0–1 | 154 (55.6) | 28 (35.4) | 126 (63.6) | < 0.001 |
| ≥ 2 | 116 (41.9) | 46 (58.2) | 70 (35.4) |  |
| Unknown | 7 (2.5) | 5 (6.3) | 2 (1.0) |  |
| Cytogenetics and molecular abnormalities |  |  |  |  |
| NPM1 | 20/125 (16.0) | 2/27 (7.4) | 18/98 (18.4) | 0.240 |
| FLT3-ITD | 18/125 (14.4) | 4/27 (14.8) | 14/98 (14.3) | 1.000 |
| Complex karyotype | 58/263 (22.1) | 22/71 (31.0) | 36/192 (18.8) | 0.034 |
| Cytogenetics and molecular risk status |  |  |  |  |
| Favorable | 36 (13.0) | 7 (8.9) | 29 (14.7) | 0.493 |
| Intermediate | 133 (48.0) | 36 (45.6) | 97 (49.0) |  |
| Poor/adverse | 94 (33.9) | 28 (35.4) | 66 (33.3) |  |
| Unknown | 14 (5.1) | 8 (10.1) | 6 (3.0) |  |
| Lab data, median (IQR) |  |  |  |  |
| Bone marrow blast, % | 80 (40–90) | 80 (53–90) | 80 (40–90) | 0.087 |
| Presence of blasts in peripheral blood | 182/275 (66.2) | 59/79 (74.7) | 123/196 (62.8) | 0.059 |
| White blood cell count, /ul | 5,830  (1,890–36,600) | 16,890  (2,400–52,910) | 4,845  (1,800–24,950) | 0.012 |
| Absolute neutrophil count, /ul | 1,254.4  (325.0–5,072.0) | 1,728.4  (517.3–9,423.0) | 1,069.3  (296.0–4,567.5) | 0.066 |
| Hemoglobin, g/dl | 8.4 (7.4–9.5) | 8.3 (7.4–9.7) | 8.4 (7.5–9.5) | 0.884 |
| Platelets, /ul | 50,000  (26,000–86,000) | 46,000  (23,000–76,000) | 52,000  (27,000–88,000) | 0.299 |
| eGFR | 65.2 (45.9–82.8) | 55.1 (30.9–78.3) | 67.0 (50.3–83.9) | 0.001 |

AML, acute myeloid leukemia; AML-MRC, acute myeloid leukemia with myelodysplasia-related changes; APL, acute promyelocytic leukemia; ECOG, Eastern Cooperative Oncology Group performance; eGFR, estimated Glomerular filtration rate; IQR, interquartile range

**Supplemental Table 2 Comparisons between different scoring systems using our elderly AML cohort**

| **Authors, journal, and publication year** | **Risk strata** | **Level** | **HR (95% CI)** | ***P* value** | **AIC** | **BIC** | **Harrell C statistics** |
| --- | --- | --- | --- | --- | --- | --- | --- |
| Malfuson JV, *et. al. Haematologica* 2008 | Prognostic index |  | 1.00 (1.00–1.01) | 0.101 | 705.27 | 707.46 | 0.60  (0.53–0.67) |
|  | Simple Decisional Index | 0 | reference |  | 736.09 | 740.59 | 0.63  (0.57–0.68) |
|  |  | 1 | 3.03 (1.72–5.32) | < 0.001 |  |  |  |
|  |  | 2 | 3.19 (1.49–6.81) | 0.003 |  |  |  |
| Wheatley K, *et. al. British Journal of Haematology* 2009 | Index | Good | reference |  | 688.86 | 693.24 | 0.63  (0.58–0.69) |
|  |  | Standard | 0.89 (0.29–2.76) | 0.839 |  |  |  |
|  |  | Poor | 3.43 (1.47–7.96) | 0.004 |  |  |  |
|  | Simplified risk score | Good | reference |  | 694.19 | 698.57 | 0.62  (0.57–0.66) |
|  |  | Standard | 1.20 (0.37–3.94) | 0.759 |  |  |  |
|  |  | Poor | 3.49 (1.40–8.71) | 0.008 |  |  |  |
| Kantarjian H, *et. al. Blood* 2010 | No. of adverse factors | 0 | reference |  | 679.96 | 686.53 | 0.69  (0.62–0.75) |
|  |  | 1 | 1.70 (0.75–3.84) | 0.204 |  |  |  |
|  |  | 2 | 3.35 (1.53–7.31) | 0.002 |  |  |  |
|  |  | ≥ 3 | 7.08 (3.22–15.58) | < 0.001 |  |  |  |
| Ramos F, *et. al. Leukemia Research* 2015 | Original ALMA score | 0 | reference |  | 664.17 | 668.49 | 0.64  (0.58–0.70) |
|  |  | 1–2 | 1.63 (0.22–11.95) | 0.631 |  |  |  |
|  |  | 3–4 | 4.92 (0.67–35.99) | 0.117 |  |  |  |
|  | European ALMA score | 0 | reference |  | 701.10 | 705.47 | 0.59  (0.53–0.65) |
|  |  | 1–2 | 2.10 (0.75–5.85) | 0.156 |  |  |  |
|  |  | 3–4 | 3.89 (1.32–11.43) | 0.014 |  |  |  |
| Walter RB, *et. al*. *Journal of Clinical Oncology* 2011 | TRM score | 0–3 | reference |  | 792.39 | 797.00 | 0.60  (0.54–0.66) |
|  |  | 4–6 | 1.49 (0.64–3.46) | 0.352 |  |  |  |
|  |  | ≥ 7 | 2.76 (1.24–6.14) | 0.013 |  |  |  |

HR, hazard ratio; CI, conﬁdence interval; AIC, Akaike information criterion; BIC, Bayesian information criterion; TRM, treatment-related mortality
